# Volatile Anaesthetic Depression of the Carotid Body Chemoreflex-Mediated Ventilatory Response to Hypoxia: Directions for Future Research

**DOI:** 10.1155/2014/394270

**Published:** 2014-04-06

**Authors:** J. J. Pandit

**Affiliations:** Nuffield Department of Anaesthetics, John Radcliffe Hospital, Oxford OX3 9DU, UK

## Abstract

In assessing whether volatile anaesthetics directly depress the carotid body response to hypoxia it is necessary to combine in meta-analysis studies of when it is “functionally isolated” (e.g., recordings are made from its afferent nerve). Key articles were retrieved (full papers in English) and subjected to quantitative analysis to yield an aggregate estimate of effect. Results from articles that did not use such methodology were assessed separately from this quantitative approach, to see what could be learned also from a nonquantitative overview. Just 7 articles met the inclusion criteria for hypoxia and just 6 articles for hypercapnia. Within these articles, the anaesthetic (mean dose 0.75, standard deviation (SD) 0.40 minimum alveolar concentration, MAC) statistically significantly depressed carotid body hypoxic response by 24% (*P* = 0.041), but a similar dose (mean 0.81 (0.42) MAC) did not affect the hypercapnic response. The articles not included in the quantitative analysis (31 articles), assessed qualitatively, also indicated that anaesthetics depress carotid body function. This conclusion helps direct future research on the anaesthetic effects on putative cellular/molecular processes that underlie the transduction of hypoxia in the carotid body.

## 1. Introduction

Hypoxia is damaging to the body and for any sustainable period if severe, incompatible with life. Many if not all of the technical and monitoring aspects of the clinical practice of anaesthesia are dedicated to preventing hypoxia. Approximately 3 million general anaesthetics are delivered each year in the United Kingdom alone [1]. Postoperative complications that render patients vulnerable to hypoxaemia are common, such as atelectasis [2] and airway obstruction [3]. Normally acute hypoxaemia is detected by the carotid bodies, generating neural afferent signals to the central nervous system respiratory control mechanisms. The result of this reflex loop is an increase in minute ventilation; the acute hypoxic ventilatory response (AHVR). However, volatile anaesthetic agents depress the hypoxic response at doses that persist well into the postoperative phase of anaesthesia [4].

Herein lies the clinical problem: a commonly encountered complication (hypoxia) coincides with the normally protective mechanisms being obtunded. Even at very low doses (<0.2 minimum alveolar concentration, MAC) the degree of depression is ~50%; at higher doses of ~1 MAC the ventilatory hypoxic response is virtually abolished [5–14]. Thus, even at sedative doses (i.e., levels that prevail for some hours after surgery) [4], patients have severely blunted protective chemoreflex responses, and this is clearly of clinical importance as patients are at risk of perioperative hypoxaemia. What is unclear is the precise site in the chemoreflex pathway (from carotid body glomus cell to integrative sites in the brain) at which anaesthetics might exert this depressive action.

Such questions are important because they concern the wider issue of “oxygen sensing.” Most body tissues suffer impaired function or harm during hypoxic exposure, but the carotid body is among the few organs that shows an* adaptive response* (with the other organs being pulmonary arterioles and the juxtaglomerular apparatus of the kidney) [15]. For a comprehensive review of the role of the carotid bodies in chemoreflex control, see Whipp and Wasserman [16]. In other words, whereas the metabolic activity of all other tissues such as cardiac and neuronal, is reduced by exposure to hypoxia, the activity of the carotid body and of the other two tissues mentioned above increases such that the carotid body glomus cells generate an intracellular calcium, Ca^2+^, transient [15]. Thus, glomus cells can be safely cultured in a hypoxic environment (e.g., 2% O_2_) for several days, an insult which would kill other tissues [17]. Insight into these adaptive mechanisms might enable their exploitation for therapeutic benefit, in terms of protection against hypoxia. More specifically, discovering the basic mechanisms by which some anaesthetics are less depressive on the hypoxic response would help define the favourable properties of these agents at cellular/molecular level.

Although it is self-evident that anaesthetics have actions on the brain and this is how they cause hypnosis (narcosis), in fact much evidence suggests that, with respect to the hypoxic chemoreflex, their main effect may instead be at carotid body level. In humans, anaesthetics at low dose selectively depress the hypoxic but not the hypercapnic ventilatory response, implying (as discussed in Section 4) an action in the chemoreflex pathway at a site before the two stimuli—hypoxia and hypercapnia—have integrated (i.e., at the carotid body) [9, 18–20]. At a cellular level, Buckler et al. have described a potassium (K+) channel in the carotid body glomus cell which is sensitive to both hypoxia and halothane, offering a plausible single mechanism within the carotid body for the human effects described [21].

There are many relevant questions we might pose in relation to this topic: does the experimental method of inducing hypoxia in research studies (i.e., rapidly as a step input using computer-controlled technologies or more slowly as a ramp input using older methods such as rebreathing) influence the results that are obtained in studying anaesthetic effect on the chemoreflex? Does the volunteer's or patient's state of arousal (i.e., awake, sedated, stimulated by noise, etc.) influence the results? Where in the chemoreflex pathway do anaesthetics act (i.e., do they influence the hypoxic chemoreflex peripherally at the carotid body or more centrally in the brain)? Can we explain in cellular or molecular terms the mechanism by which anaesthetics depress the chemoreflex? Do different anaesthetic agents depress the hypoxic response differentially, or equally? Are there clinically relevant consequences of the anaesthetic depression of chemoreflex? In this review, we will focus on the last two and ask primarily where anaesthetics act within the chemoreflex pathway.

Perhaps the most direct experimental means of establishing the site of action is simply to measure the response of functionally isolated animal carotid bodies to hypoxia/hypercapnia. This can only be undertaken in animal studies and several such studies have been undertaken. Yet the last major review into this specific question drew an unambiguous conclusion, in which “*the chemosensitivity of the carotid body chemoreceptor seems not to be influenced by volatile inhaled anesthetics*” [22]. This assertion implies that, rather than a primary effect at the carotid body, any depressive effect of anaesthetics occurs instead at a site more centrally in the hypoxic chemoreflex loop (i.e., in the brain)—as that review argued, “*The depression of hypoxic ventilatory response during anesthesia must therefore originate within the central nervous system*” [22]. If this conclusion was true, then there would be little reason to explore volatile anaesthetic action on the carotid body. But this would seem to be at odds with the aforementioned lines of evidence [9, 18–23].

Before accepting that earlier review's conclusions uncritically, it is important to appreciate some of its limitations. First, it was not* systematic*; that is, it did not define explicit search strategies or specific criteria for accepting or rejecting retrieved papers. Rather it was a* subjective* assessment of the data in which personal as opposed to objective weighting was given to the evidence. It is now recommended that any review should be objective and systematic, rather than subjective [23, 24]. Second, the review referred to just 9 animal reports and it included one abstract. This may indeed have reflected the true paucity of data in the field, or it may have indicated that the nonsystematic approach had failed to retrieve all the available evidence and further inclusion of an abstract is not recommended for quantitative reviews. Third, the synthesis of the data from the retrieved reports was not quantitative: the conclusion that the carotid bodies are “minimally affected” is very much a qualitative statement and does not tell the reader the magnitude of effect. The available data might have been used to estimate the likely degree of depression of hypoxic response in more quantitative terms. Finally, Eriksson did not discuss any evidence related to carotid body* hypercapnic* response [22].

One method of resolving these contradictions is by systematic search of the literature for relevant material and employing a quantitative synthesis of the results retrieved.

The starting point of this review is therefore to address the very simple question: what is the evidence that volatile anaesthetics influence the hypoxic ventilatory response by an action at the carotid body? In providing an answer, we will also discuss the wider importance of studies of oxygen sensing on anaesthetic practice and research.

## 2. Methods 

Using the Bodleian Library services at Oxford University (EMBASE, MEDLINE/PubMed, Ovid), an electronic search was conducted (1950–2012) using a combination of appropriate search terms (e.g., “an(a)esthetics”, “ventilation”, “control of breathing”, “carotid body”, “chemoreflex”). This was supplemented by a manual search of the reference lists of initially retrieved papers. The reference lists from the published papers, reference articles, and correspondence retrieved by this initial search were used to search manually the papers in the listed journals. Each of the authors involved in the publications retrieved was also searched electronically using the above databases to assess whether they had been involved in other relevant publications. The journals manually searched were:* British Journal of Anaesthesia, Anesthesiology, Anaesthesia, Anesthesia and Analgesia, Canadian Journal of Anaesthesia, Acta Anesthesiologica Scandinavica, Journal of Physiology, Journal of Applied Physiology, and Japanese Journal of Physiology and Respiration Physiology and Neurobiology*. In addition, abstracts from the following meetings were searched: American Society of Anesthesiology Annual Meeting (published on-line in* Anesthesiology*); International Anesthetic Research Society Meeting (published online in* Anesthesia and Analgesia*); Anaesthetic Research Society (UK) Meeting (published as symposium proceedings and in* Anaesthesia*); Triennial International Meeting on Modelling and Control of Breathing (published in* Advances in Experimental Medicine and Biology*); Symposium on the Neural and Chemical Control of Breathing, Leiden University, The Netherlands (published as a monograph). Finally, the following doctoral theses were also searched: Pandit (1993)* The Effects of Exercise on the Chemical Control of Breathing in Man,* and Nagyova (1997)* Ventilatory Response to Hypoxia in Humans* (both at the Bodleian Library, Oxford); Dahan (1990)* The Ventilatory Response to Carbon Dioxide and Oxygen in Man*, and van den Elsen (1997)* Influence of Low Dose Anaesthetic Agents on Ventilatory Control in Man* (both at Leiden University, The Netherlands). The Cochrane Controlled Trials Register (http://www.thecochranelibrary.com/view/0/index.html) was also searched in which, although is a register for human trials, relevant sources may have been found in reference lists.

### 2.1. Inclusion Criteria and Assessment of Quality

Articles that met the following criteria were considered relevant: (a) the article was published as a full paper: abstracts and data in letters or case reports were excluded; (b) the article was published in English (or an English cotranslation was provided in the text); (c) the article concerned animal investigation (i.e., not human physiology); (d) volatile anesthetics commonly used in recent clinical practice were used in the article (i.e., halothane, enflurane, isoflurane, sevoflurane, and desflurane).

### 2.2. Quantitative Analysis

In the quantitative analysis, the plan was to include only those articles whose experimental protocol* functionally isolated* the carotid body and so enabled any anaesthetic effect to be located to this organ. For example, if a study exposed the whole animal to anaesthetic but simply then recorded from the phrenic nerve during hypoxia or hypercapnia, this did not functionally isolate the carotid body, since in such a protocol any observed anaesthetic effect might be primarily on the brain. However, a study that exposed the whole animal to anaesthetic but recorded from the carotid sinus nerve did measure the direct drug effect on the carotid body. The following general experimental preparations are suitable.

(a) Any study in which recordings were taken from the carotid sinus nerve (the afferent nerve of the carotid body; anaesthetic being administered either to an isolated, separately perfused carotid body or to the whole animal).

(b) Any study in which the carotid body was physically isolated and so could be separately perfused and so separately exposed to anaesthetic and/or hypoxia and/or hypercapnia. In this experimental procedure, recordings might be taken either from the carotid sinus nerve or from the phrenic nerve or from the minute ventilation of the animal. Such methods of isolation might include methods that separately perfused the carotid body from the systemic circulation, or artificial brainstem perfusion techniques that separated the brain's circulation from that of the body (including from that of the carotid body).

It is important to exclude from quantitative analysis all abstracts, letters, case reports, non-English work, and studies on historical agents (e.g., ether, chloroform, methoxyflurane, and cyclopropane). Also, because the focus of enquiry is on volatile agents, also excluded are reports on nitrous oxide and xenon (both gases) or any intravenous agents (in fact, none came to light).

For articles included in the analysis, the following was recorded: the animal species (and number) studied; whether neuromuscular blockade was used during the experiment; the anaesthetic used and its dose. If an article presented only a range of doses and did not specify the exact dose administered, the middle of the range of doses was used. Where a published* article* contained data from more than one agent, or the same agent at more than one dose, or had examined more than one species of animal, the data for each agent, each dose, or each species was regarded as a separate* study* for analysis.

The outcome measure of interest was simply the proportion by which anaesthetic depressed the measured response to hypoxia or hypercapnia in the selected study. For example, in studies measuring activity of the carotid sinus or phrenic nerves, the response was in impulses·s^−1^ of the relevant nerve. For studies measuring the animal's minute ventilation, the response was in L·min^−1^. In combining standardised anaesthetic effects for hypoxia or hypercapnia of more than one study, we weighted by the size of the study (i.e., number of animals used), and statistical analysis was undertaken on these weighted means [25].

In summary, each published* article* yielded one or more separate* studies*. Each* study *yielded an* anaesthetic effect *for hypoxic response and/or an* anaesthetic effect *for hypercapnic response. The standardised anaesthetic effects for all studies were averaged (with weighting) and analysis was undertaken on these weighted means [25].

Analysis of variance (ANOVA, SPSS for Windows version 10.0, SPSS Inc., Chicago, IL, USA) was performed on the data [26]. The values for standardised anaesthetic effects for hypoxic or hypercapnic response, respectively, were used as the “response” term for the ANOVA. There were three fixed factors: “agent” (three levels, one for each agent), species (two levels, one for each species studied), and “neuromuscular blockade” (two levels, indicating whether or not blockade was used in the experiment). If ANOVA suggested any statistically significant effects, these were further explored using post hoc *t*-tests with Bonferroni correction at the appropriate level. A value of *P* < 0.05 was taken as statistically significant.

### 2.3. Qualitative Analysis

In addition to quantifying anaesthetic effect, it was appreciated that any papers rejected from the quantitative part of the study may contain useful information. It is important to include these in a qualitative commentary to assess if their conclusions supported or not any conclusions of quantitative analysis.

## 3. Results

**Figure 1:**
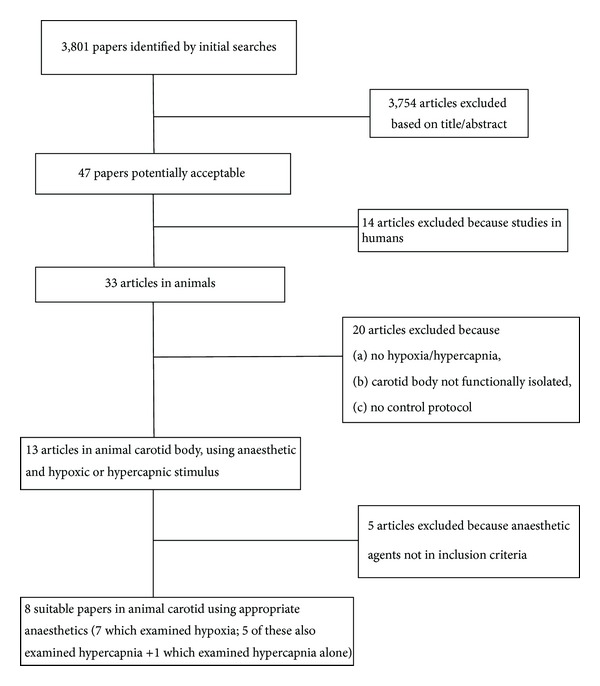
Flow diagram showing the selection of articles for analysis.

Figure 1 indicates the flowchart for retrieval and inclusion of papers. After promptly excluding a number of articles retrieved by the initial search, another 14 articles were excluded which were conducted in humans or had used intravenous agents [5–15, 18–20, 27–30] and 20 articles that did not use hypoxia or hypercapnia as stimuli or had used experimental techniques that did not functionally isolate the carotid body or used no control (i.e., without anaesthetic) protocols [31–50]. Five articles examined agents not in the original inclusion criteria [51–55]. This left just 7 articles from which data could be used to assess hypoxic responses [56–62] and 6 to assess hypercapnic responses [56, 57, 60–63].

### 3.1. Anaesthetic Effect on Hypoxic Responses

From 1968 when one study examined one animal (at two doses of halothane) [56], there was no publication in this field until 1982 [57]. The 7 articles yielded 16 studies for analysis (Table 1). Six of these have come from just one publication [60]. The most recent publication in this field was ~14 years ago [59]. Only three agents have been examined: halothane (10 studies), isoflurane (4 studies), and enflurane (2 studies). Only the cat or rabbit has been used, and all but three studies used neuromuscular blockade during the experiment.

**Figure 2:**
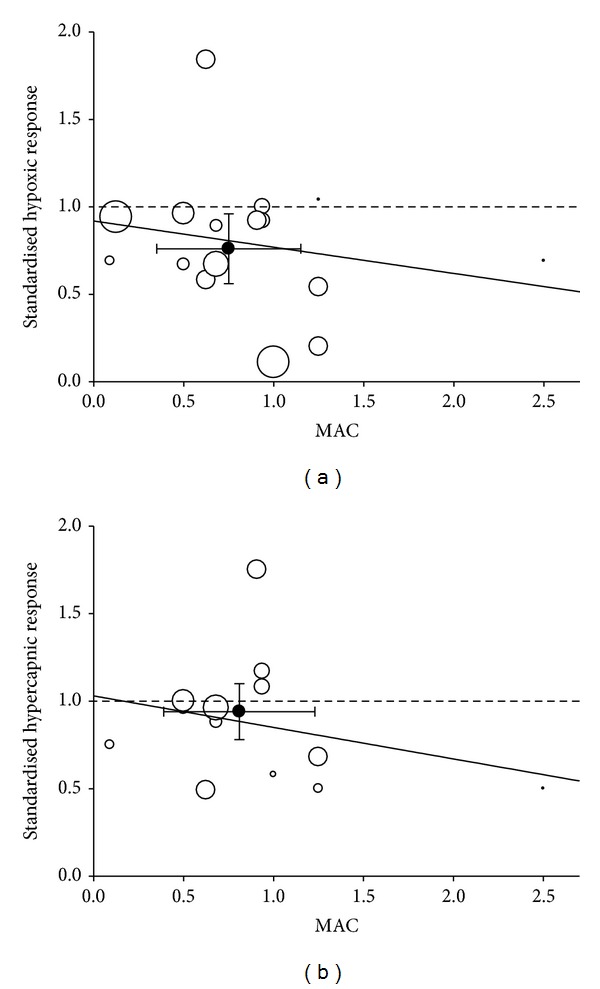
(a) Standardised anaesthetic effect on hypoxic response for each of the studies in Table 1. The horizontal dashed line indicates that at a value of <1.0 for standardised anaesthetic effect implies depression of hypoxic response by agent; a value of 1.0 indicates no effect and a value >1.0 suggests stimulation by agent. Each study is plotted by size (diameter) of the symbol (smallest *n* = 1; largest *n* = 10). The dark symbol represents the weighted mean value and error bars ±95% confidence intervals. (b) Standardised anaesthetic effect on hypercapnic response for each of the studies in Table 1. Within each panel the solid line indicates the best fit line (linear weighted least squares regression). The best fit equation for hypoxia is *y* = −0.1479*x* + 0.9194; that for hypercapnia is *y* = −0.1762*x* + 1.03.

The mean (standard deviation, SD) dose of anaesthetics used in all studies combined was 0.75 (0.40) MAC. The weighted mean anaesthetic effects for the agents were similar: halothane 0.73 (0.53), enflurane 0.85 (0.23), and isoflurane 0.79 (0.11), *P* = 0.96. For all agents combined, the weighted anaesthetic effect was 0.76 (0.42), 95% confidence interval 0.56–0.96, indicating that on average anaesthetics significantly depressed carotid body hypoxic response by 24% (*P* = 0.041) ( Figure 2).

Figure 2 also shows the effects of increasing anaesthetic dose on responses to hypoxia showing a modest dose-dependent decline in response.

For completeness we conducted a subgroup analysis to explore the potential effect of neuromuscular blockade and species on the effect of anaesthetic, However, neither was significant (ANOVA: *P* = 0.620 and *P* = 0.296 resp., when factors considered random). Thus, notwithstanding the small size of subgroups, the mean result of studies using neuromuscular blockade (0.72 (0.23), *n* = 3) was similar to that of studies using no blockade (0.76 (0.47), *n* = 13); and the mean result of studies in cats (0.77 (0.47), *n* = 11) was similar to that of studies in rabbits (0.83 (0.15), *n* = 5).

### 3.2. Anaesthetic Effect on Hypercapnic Responses

For hypercapnia, our search yielded the same papers as for hypoxia and we additionally located one article that examined peripheral CO_2_ (but not hypoxic) sensitivity using an artificial brain perfusion technique at two doses of halothane in cats [57, 58].

Table 1 also presents the results for the 13 studies (from 6 articles) in hypercapnia. The overall result was that anaesthetic at mean dose 0.81 (0.42) MAC did not significantly affect the hypercapnic response (mean anaesthetic effect 0.94 (0.35), 95% confidence interval 0.76–1.14, *P* = 0.165) (Figure 2). There was insufficient data to undertake any subgroup analyses on the hypercapnic responses. Figure 2 also shows a modest dose-dependent decline in response.

## 4. Discussion

In answer to the primary question addressed, the main conclusion of the quantitative review is that volatile anaesthetics moderately but significantly depress the carotid body response to hypoxia but they do not, however, influence carotid body response to hypercapnia (although there is a dose-dependent depression of the CO_2_ response such that at high doses this may be suppressed, an effect which could occur at the carotid bodies (Figure 2)). These results are consistent with a large body of human literature [5–15], but at odds with the conclusion of the last major review in the field [22].

The finding that volatile anaesthetics impair carotid body sensing of hypoxia is important. Undoubtedly, the primary site of the* hypnotic* (narcotic) action of anaesthetics is the brain. It is therefore tempting to assume that therefore the site of anaesthetic depression of the hypoxic chemoreflex must also be centrally in the brain. However, since the brain is* upstream* of the carotid body, anaesthetic agents can only suppress the chemoreflex centrally if information is arising peripherally from the carotid bodies and transmitted to the brain. If, as is shown in this review, anaesthetics have obtunded the carotid body response then this is one situation where, perhaps counter-intuitively, the peripheral action of agents is more important than any central action. The importance of this is arguably further underlined by Smith et al. [64] who argue that the peripheral and central chemoreceptors are not independent, but that the degree of peripheral stimulation critically influences central sensitivity. The notion of peripheral-central interaction has also been assessed in humans [65, 66]. If this is the case, then the peripheral depressive effect of volatile anaesthetics as shown in this review becomes a more important phenomenon for respiratory control. However, an assumption has been made in this review that all or most of the ventilatory response to hypoxia is confined to the carotid bodies. Although that appears correct for all the species examined in this review, Curran et al. [67] have reported the finding that central neurones are sensitive to hypoxia in dogs. The extent to which this is a wider phenomenon that is unclear.

We discuss below the wider implications of the main result.

### 4.1. Limitations of Nonsystematic Reviews

A previous review included consideration of studies whose methodology did not functionally isolate the carotid body, yet conclusions were nonetheless drawn about anaesthetic-carotid body interaction [22]. For example, the studies of Gaudy et al. [35, 36], Koh and Severinghaus [42], and Stuth et al. [45, 46] were explicitly quoted in support of a conclusion that there must be no anaesthetic effect on the peripheral chemoreceptor. In contrast, the systematic approach conducted here excluded these studies from quantitative analysis because in these reports, anaesthetic had been administered to the whole animal while, respectively, recording phrenic nerve activity, minute ventilation, or blood gases (none of which functionally isolate the carotid body). These methodologies are not therefore able to robustly distinguish an anaesthetic effect on carotid body function as opposed to an effect at another, more central site.

In the earlier review [22], it was stated that results of all animal studies in this field were “*uniform*”; that is, all papers had hitherto suggested little or no anesthetic effect on the peripheral chemoreceptor. The results here do not support this contention. Table 1 shows a tenfold difference in the crude standardised hypoxic responses (range 0.11–1.04), which superficially indicates considerable heterogeneity, and Figure 2 confirms this graphically. Nevertheless, the confidence intervals of the analysis are sufficiently narrow to reflect a statistically significant anaesthetic effect when the data were combined. One possible reason for some relatively disparate results may be if the “true” dose-response relationship for anesthetic effect on hypoxic response is quite steep for doses between ~0.5 and 2 MAC. Then, small errors in dosing or measurement could have quite large effects on anaesthetic effect.

The “uniformity” for lack of effect previously suggested by Eriksson [22] is challenged by the comments made by authors in individual papers. While Joensen et al. stated that “*1 MAC isoflurane does not depress the hypoxic response of rabbit carotid body chemoreceptors*” [59], Ide et al. in contrast stated: “*the hypoxic ventilatory response is depressed by halothane through a peripheral effect at the carotid body*” [58]. Davies et al. found that “*halothane has a direct effect on carotid body chemoreceptor mechanisms*” [57]. Making a somewhat less decisive statement, Ponte and Sadler opined: “*it is most likely that volatile anaesthetics exert a direct action upon the chemoreceptor mechanism*” [61], van Dissel et al. “*could not exclude*” an effect of halothane on the peripheral chemoreceptor [62], and Morray et al. concluded that the anesthetic effect was “*at least partially explained by an effect on peripheral chemoreceptors*” [60]. Thus, the consensus of the majority of authors actually favors the notion that anaesthetics depress carotid body function (Table 1). Furthermore, as discussed below, this is also consistent with more recent studies that find depressive effects of anesthetic on molecular structures within the carotid body putatively involved in the transduction process.

### 4.2. Strengths of a Quantitative Review

The quantitative nature of the analysis presented here enables a more precise estimate of anaesthetic effect to be described than can a more subjective review of the literature. As compared with the previous nonsystematic approach [22], the systematic search strategy was also able to locate an additional study by Morray et al. [60] and by Ide et al. [58] both of which had found depression of carotid body function by anaesthetic (Table 1).

It is pertinent here to comment on the article of Ponte and Sadler [61]. They reported that a “*chemodepressant effect of anaesthetics was absent at PaO*_*2*_ *values <5.3 kPa.*” This statement appears to have been taken at face value and repeated by others in review [22], but in fact there appear to be no data in the paper of Ponte and Sadler to support this assertion. On the contrary, their graphical data suggests that the greatest magnitude of depression by anaesthetic occurred at the lowest level of hypoxia (~5.3 kPa) [61].

Similar reappraisal of published data applies also to the article of Joensen et al., who concluded in their study that carotid body chemoreceptors “*were not depressed by isoflurane*” [59]. However, their data indicate that 0.1% isoflurane depressed hypoxic response by 30% (Table 1). Even if the control data for the 3 cats in their study that were exposed to 0.1% isoflurane are used alone (rather than the control data for all 6 cats combined), the hypoxic response is depressed by 13%, but this does not alter the overall weighted mean value, which remains 0.76 ±  0.42 (24% depression), nor changes the statistics of Table 1. So, regardless of the method of calculation used for their published data, these authors' stated conclusion is at odds with their own data, which finds a clear depression of the carotid body hypoxic response by anaesthetic. Furthermore, the authors found a higher dose of 1% isoflurane in the same article that was paradoxically less depressive (just 8% depression) than 0.1% (whichever calculation is used for the latter). The authors left this paradox unresolved. It is only with a formal quantitative methodology that these intricacies are revealed (Table 1).

While a quantitative review arguably offers a perhaps more objective analysis than does a subjective review, its ultimate utility depends almost entirely on the quality of the original studies included in the analysis. The method of weighting studies by size and statistical methodology can only in part compensate for any shortcomings in the original work.

By excluding agents such as chloroform, cyclopropane, and ether, important scientific data may be missed, but the clinical relevance of such agents is now very limited.

One limitation to quantitative analysis is that the true relationship between ventilation (or carotid sinus nerve or phrenic nerve discharge) and partial pressure (PO_2_) is nonlinear [68]. So the proper way of assessing the hypoxic response is using a range of values for PO_2_. However, none of the original papers has been so comprehensive. With this shortcoming of the original studies in mind, it is appropriate to choose to use data from a single point (i.e., the response to a hypoxic stimulus of PO_2_ ~6.6 kPa, with background PCO_2_ ~5.3 kPa) for all articles. Justification for this is that this PO_2_ stimulus is a common or even “standard” hypoxic stimulus in human studies [9], and all animal studies retrieved by us used at least this level of hypoxia. Nonetheless, if experimentally one wished to delineate the precise estimate of effect, then one should properly attempt to map responses over a wider range of hypoxic stimuli.

### 4.3. Quality Ranking of the Articles Retrieved

The methodology used in this review makes it possible to rank the articles according to their quality (Table 1, last column). Note that this ranking relates specifically to the question addressed in this review, and not necessarily to the wider value of the article as a contribution to science, or indeed, to the original purpose of the article when it was written [69, 70]. Articles are ranked lower in the order if they used very few or potentially immature animals (Biscoe and Millar [56], Morray et al. [60]), if their results were internally inconsistent (Joensen et al. [59]), or if they did not control or specify the anaesthetic dose (Ponte and Sadler [61]) (Table 2). The methodologies of Ide et al. [58] and Davies et al. [57] are very persuasive (and the results of these two studies themselves seemed consistent with each other), and of van Dissel et al. [62], although this last is ranked a little lower than Ide et al. or Davies et al. simply because it did not specify an exact anaesthetic dose.

### 4.4. The Effects of Neuromuscular Blockade

Nondepolarising neuromuscular blocking drugs can blunt the carotid body response to hypoxia [22, 71–73]. Acetylcholine released within the carotid body, acting on nicotinic receptors, is directly implicated in the hypoxic transduction process but we were unable to demonstrate any independent influence of neuromuscular blockade on the aggregate results.

Joensen et al. [59] and Eriksson [22] argued that in animal studies where neuromuscular blockade was used, this—and not the anaesthetic—had caused the depression of hypoxic response, thus confounding the proper interpretation of results. However, we found that since studies normally employ a control protocol without anaesthetic, any separate depressive effect of neuromuscular blockade on hypoxic response was presumably constant for both control (i.e., without-anaesthetic) and test (i.e., with-anaesthetic) protocols. In other words, the majority of studies that demonstrate depression of carotid body hypoxic response by anaesthetic, despite the presence of neuromuscular blockade, are clearly showing some anaesthetic effect* per se*. Furthermore, studies like that of van Dissel et al. [62] avoided neuromuscular blockade (the animals breathed spontaneously) but these authors nonetheless found a large depressive effect of anaesthetic on the peripheral chemoreceptor of ~50% (Table 1). We conclude that while neuromuscular blocking drugs might independently inhibit the carotid body, they may not necessarily mask the action of anaesthetic.

### 4.5. Species Differences

Only two species, cat and rabbit, have been studied and this is too few to make generalisations about “animals” in general. Furthermore, it is now apparent that even within a species, genetic factors can play an important role. Weil et al. demonstrated that the ventilatory hypoxic response of spontaneously hypertensive (SHR) rats was seven times higher than that of the Fischer 344 (F344) strain [74]. Similarly in mice, Yamaguchi et al. described structural and functional differences in the carotid bodies of two strains (DBA/2J and A/J) [75]. However, any additional interactions of these genetic influences with anaesthetic effect remain unexplored. Similar genetic effects may underlie the observed differences in human hypoxic ventilatory sensitivity [76, 77], and it is unclear if overall any between-species variation is less than or greater than any within-species variation.

### 4.6. Hypercapnic Responses: Comparison of Animal and Human Data

**Figure 3:**
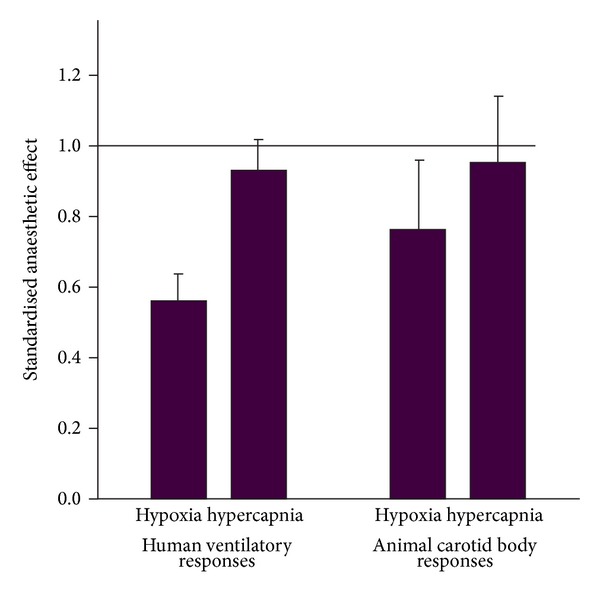
The mean effect of low dose (<0.2 MAC) anaesthetics on hypoxic and hypercapnic ventilatory responses in humans (values taken from data presented in references [5, 13]) and the data for animal carotid bodies (from this study). The smaller the value for standardised anaesthetic effect, the greater the depressive action of anaesthetic. For both animal carotid body and human ventilation, anaesthetics significantly depress hypoxic but not hypercapnic response. The error bars represent 95% confidence intervals. The horizontal line represents the line of unity (i.e., no effect of anaesthetic).

The lack of anaesthetic effect on hypercapnic responses presented here in aggregate form is novel, and there does not seem to have been a previous review of anaesthetic effect on carotid body CO_2_ response. In contrast to effect on hypoxic response, there was no significant effect of anaesthetic on CO_2_ response and this resembles what is found in human studies (Figure 3) [18, 20].

### 4.7. Consistency of Conclusions with a Qualitative Analysis

The quantitative part of this review forms just one part of a wider assessment of the available evidence. Notably, rejected from* quantitative* analysis were those experimental techniques (in both humans and animals) that estimated carotid body function more indirectly. Now, some of these papers can be considered in a more* qualitative* manner. In interpreting such studies it is important to appreciate three axioms. First, the carotid body contains separate and specific transduction mechanisms for hypoxia and hypercapnia [78, 79]. Second, each afferent nerve carries information about both CO_2_ and hypoxia and is not dedicated to one or the other (thus the brain cannot distinguish the type of stimulus involved in activation, but only its magnitude) [79]. Third, the synergistic effect of these two stimuli occurs at the carotid body, not more centrally in the brain [79, 80]. Thus, if a drug such as an anaesthetic affects one response (e.g., hypoxia) more than it does the other (e.g., hypercapnia), it is interpreted to suggest a peripheral rather than a central effect of that drug (because a central action could not specifically influence just the one response in isolation). In the light of these axioms we have the following.

*(1) Evidence That Anaesthetics Act on the Peripheral Chemoreflex in Animals*. In dogs, Weiskopf et al. found that 1.1% halothane impaired the ventilatory response to hypoxia to a greater extent than to CO_2_, and also that it attenuated the synergistic effect of hypoxia and CO_2_ on ventilation [49]. In vagotomised dogs, Stuth et al. found a dose-dependent reduction in phrenic nerve activity to halothane (0.5–2 MAC) in response to rapid (~1.5 s) boluses of 100% CO_2_—infused saline into carotid arteries, a stimulus likely to activate the carotid bodies rather than the central chemoreceptors [45–47]. Dahan et al. used square-wave changes in end-tidal sevoflurane in anaesthetised cats and, after analysing responses mathematically, they found a greater reduction of peripheral than central chemoreflex gain [33].

*(2) Evidence That Anaesthetics Act on the Peripheral Chemoreflex in Humans. *First, hypoxia-driven ventilation decreases within 30 s of exposure to subanaesthetic concentrations of both halothane [5–14] and isoflurane [5–15], a time interval too short to affect the brain, but adequate to influence the peripheral chemoreceptor. Second, acute metabolic acidosis selectively activates peripheral, not central, chemoreceptors, and its effect is abolished by halothane (0.1 MAC) [5–14]. Third, if the ventilatory response to hypercapnia is analysed mathematically into fast (peripheral) and slow (central) components, it is the former that is preferentially reduced by volatile anaesthetics [23, 29]. Finally, combining results of many human studies indicates that low dose, subanaesthetic (<0.2 MAC) volatile anaesthetics blunt the ventilatory response to hypoxia [9] but not hypercapnia [18] (Figure 3), a result which is more consistent with the notion that their effect is on the peripheral chemoreceptor rather than on central mechanisms.

*(3) Evidence of Anaesthetic Action on Cellular/Molecular Processes in Carotid Body. *Buckler et al. reported that halothane blunted the response of a background K+ channel in isolated rat glomus cells to hypoxia [21]. Very recently, Pandit et al. have extended these observations to show that halothane and sevoflurane inhibit the intracellular calcium transient of these cells to hypoxia [81, 82], and that halothane and isoflurane antagonise the action of hypoxia on background rat glomus cell K+ channels measured using voltage (patch) clamp techniques [83]. Halothane, sevoflurane, and isoflurane all influence activity of human background K+ channels expressed in oocytes [84] and halothane, enflurane, and desflurane all influence K+ channel activity of Fisher rat thyroid epithelial monolayer preparations [85]. Closure of background K+ channels is probably the key step in the transduction of hypoxia by the carotid body and anatagonism of this by anaesthetics is therefore a very plausible mechanism which explains the blunting of human hypoxic ventilatory response by these agents. Finally, Karanovic et al. have reproduced in a rat model the predictions made by Pandit of human studies; namely, reporting that (a) volatile agents act on the peripheral chemoreceptor and (b) exhibit a specific order of potency for this effect as found in humans (i.e., halothane > enflurane > isoflurane > sevoflurane) [86]. 

In this review, two animal studies supported a primary central rather than peripheral effect of anaesthetics (neither of these functionally isolated the carotid body, so they were excluded from quantitative analysis). Stuth et al. found that halothane reduced hypoxic and hypercapnic phrenic nerve responses in vagotomised dogs equally rather than differentially (i.e., contrary to the result shown in Figure 3) [45–47]. This result was unusual in that it rather contradicted their own results of a contemporaneous paper [45–47] that had indicated a more peripheral halothane effect. Also, it was surprising that in all papers Stuth et al. found even in very high doses of halothane (2 MAC), hypoxic/hypercapnic responses were present: usually, responses are abolished at this very high dose. In goats, Koh and Severinghaus found that hypoxic and hypercapnic responses were similarly reduced by halothane (1.25% end-tidal), consistent with a central rather than peripheral effect [42].

## 5. Remaining Controversies as Focus for Research

**Figure 4:**
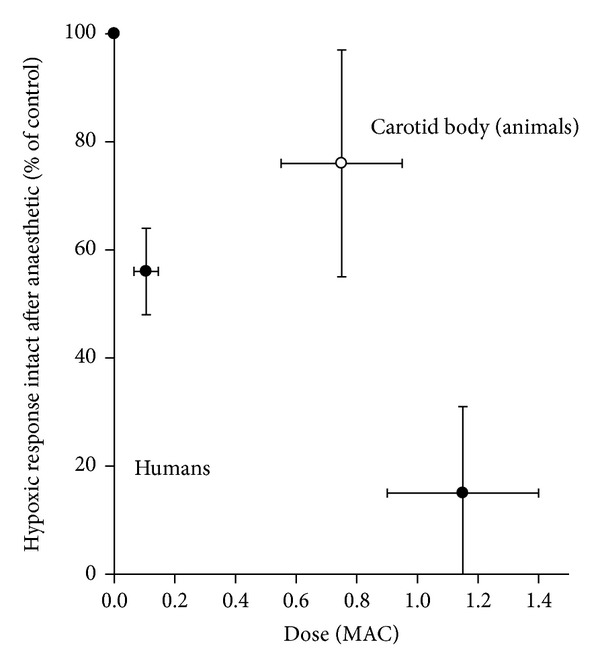
Plot of dose-response relationships between the effect of anaesthetic on hypoxic response, expressed as the % hypoxic response intact in presence of agent versus MAC for animal carotid bodies (this study from Table 1), human data at low dose (data from reference [5]), and human data at high dose (data from references [1–4, 9–11, 20, 21, 72]). The points are means (±95% confidence intervals).

Superficial comparison of the animal results presented here and the human hypoxic ventilatory response suggests that the latter is more sensitive to anaesthetics (Figure 4). The possible interpretations of this lead to potential areas of human, clinical, and animal research.

First, since many animal studies were conducted with at least some background anaesthesia (e.g., ketamine or chloralose) in the “control” condition, any additional inhibition by halothane, enflurane, or isoflurane in the “test” condition may have been underestimated, especially if drugs such as ketamine slightly blunt the hypoxic response. The “true” animal response relationship may therefore be closer to the human relationship in Figure 4. Studies are sparse on the effect of agents such as ketamine on human or animal hypoxic responses, so this is an area for further investigation (e.g., a recent review of ketamine suggested that the incidence of hypoxaemia in clinical practice may be lower with ketamine than other agents [87]).

Second, the greater apparent sensitivity to anaesthetics in humans (Figure 4) may be explained by species differences, presumably embedded in different transduction mechanisms or membrane channel subtypes in humans versus animals (which in turn are encoded by the genetic makeup of the species). Although there are recognised to be genetically-based differences between human volunteers in their hypoxic responses, Weil has observed that researchers tend only to study those volunteers with robust responses [74]. So it remains unknown if humans with more modest responses exhibit different sensitivity to the depressive effect of anaesthetics.

Third, the greater apparent sensitivity to anaesthetics in humans (Figure 4) may be explained by an additional central (i.e., brain) depressive effects of anaesthetic in human studies, of course absent from the functionally isolated carotid body studies examined here in animals. Whether different anaesthetics interact with the hypoxic stimulus in different ways at brain level and in which parts of the brain these interactions may occur are questions amenable to investigation using techniques such as functional magnetic resonance imaging (as has been suggested elsewhere [88]).

The mechanisms of oxygen (and CO_2_) sensing in the carotid body remain poorly understood but most probably involve a type 1 cell membrane channel and/or cytoplasmic, non- or mitochondrial heme containing enzymes [16, 89, 90]. Recent work suggests that one (or more) K+ channels (of the two-pore/four transmembrane segement family, termed TREK, TASK, TWIK, TRAAK, Kv, maxi-K) may initiate the O_2_-sensing cascade [91, 92]. There may be some species differences here, with perhaps TASK-like channels implicated in rats [17]; Kv channels implicated in rabbit [93].

The exact mechanism by which hypoxia closes K+ channels is unknown but may be related to their sensitivity to reactive oxygen species (ROS) and/or changes in their redox state [94]. In hypoxic conditions, halothane produces ROS, an effect which mediates its adverse effects on the liver through lipid peroxidation [95]. Halothane also increases K+ channel (especially TASK channel) conductance [21]. These two observations raised the possibility that halothane might reduce carotid body/ventilatory response to hypoxia by producing ROS. Franks and Lieb tested this hypothesis by pretreating human subjects with an antioxidant cocktail of ascorbic acid and *α*-tocopherol: consistent with the notion that halothane acts via ROS, this reversed its inhibition of the hypoxic response [92]. Teppema et al. also showed that antioxidants reverse isoflurane-induced depression of hypoxic response [96]. Furthermore, they observed a relationship between the degree to which an anesthetic is metabolized and produces ROS to the degree to which it blunts the hypoxic response [97, 98]. Thus, metabolism is the highest for halothane > enflurane > isoflurane > desflurane, and degree of depression of hypoxic response is also halothane > enflurane > isoflurane > desflurane [9]. Perhaps, though sevoflurane alone breaks this trend since its metabolism is higher than isoflurane, it is less depressive of the hypoxic response [9].

These observations relating to ROS may be extended to animals and thereby also explain some of the variability noted in this review. Dahan and Teppema have noted that ascorbic acid (an antioxidant) production varies in species and, for example, is high in the goat > rabbit > cat > human [99]. The “ROS-K+ channel hypothesis” predicts that the hypoxic response of the goat should therefore be more resistant to the depressive effects of halothane than the human and, if we accept the results of Koh and Severinghaus for goats referred to above, this does indeed seem to be the case [42]. However, the quantitative analysis presented here indicates that there are as yet insufficient data to comment on differences between cat and rabbit (Table 1). Although this “ROS-K+ channel” hypothesis is perhaps attractive for being a potential “unifying mechanism” for some of the effects described in this review, Cotten and Miller identified a number of serious problems with the theory [85]. Most notable was the fundamental deficiency that it is still not established whether hypoxia increases or decreases ROS production [95].

Furthermore, this is not the only mechanism proposed: others include that, for example, complex 1 (nicotine adenine dehydrogenase:ubiquinone oxidoreductase) and complex II (succinate ubiquinone dehydrogenase) are possibly involved in the O_2_ sensing cascade and suggested molecular targets for anesthetics [100, 101].

## 6. Conclusions

Operative and perioperative hypoxia remain a leading cause of anaesthesia-related morbidity and mortality, whether this arises from postoperative complications or airway management problems [102–104]. Hypoxia-linked problems are also a leading cause of litigation [103]. Millions of anaesthetics are administered worldwide every year (~3 million per year in the United Kingdom alone [1]) and postoperative hypoxaemia (especially nocturnal hypoxaemia) is recognised as a serious and independent risk factor for myocardial infarction, thromboembolic disease, brain injury, and confusion [105]. Patients with preexisting cardiorespiratory and neurological disease are most susceptible to the harmful effects of hypoxia, and more of these patients are presenting for surgery [106]. Because so many healthy patients undergo surgery, even the modest risk reduction will translate to large reductions in the burden on health services. Since low concentrations of volatile anaesthetics long after surgery and depress the ventilatory response to hypoxia, (an effect magnified by coadministered drugs like opiates), any intervention or combination of drugs which maintains the ventilatory response will logically be protective against hypoxaemia [107]. The provision of adequate analgesia and prevention of postoperative respiratory complications is highly relevant in hospital care, so our results will help inform strategies designed to achieve optimal outcomes.

This review underlines the importance of a systematic approach to the evidence, as opposed to a subjective approach. The main conclusions of this review can be summarised as follows.

1. Volatile anaesthetics depress the ventilatory response to hypoxia primarily by an action on oxygen sensing mechanisms within carotid body (rather than actions more centrally in the brain).
2. This is important for understanding the mechanisms of perioperative hypoxaemia, as a situation where the patient's natural defence mechanisms against hypoxia (e.g., a robust ventilatory response) are blunted.
3. Future research can fruitfully focus upon (a) the mechanisms of oxygen sensing within this organ and (b) developing agents that retain an anaesthetic action whilst avoiding actions that interfere with oxygen sensing.

This systematic review reaches a conclusion different from that of a previous analysis [22]. If the conclusion of that earlier analysis was correct—namely, that anaesthetics have a minimal effect on the carotid body—there would seem little justification for further investigating any anaesthetic effects on this organ. The consequence of the very different conclusion proposed in this review is that it becomes important to identify the cellular/molecular processes that underlie anaesthetic depression of carotid body hypoxic responses. This will in turn require further elaboration of the processes by which the carotid body senses hypoxia at cellular level. This is fundamental to understanding how the body as a whole protects itself from the harmful effects of hypoxia. The complementary human and animal studies suggested by our review will offer insights into this question.

## 7. Clinical Implications

Goodman had previously argued that reduction of AHVR by anaesthetics would not result in harm, because at anaesthetic doses of drug, an anaesthetist was* always *on hand to provide care. He suggested it was mechanical airway obstruction, rather than reduced ventilatory response to hypoxia, that causes harm [108]. This view has not stood the test of time. Postoperative hypoxia is in fact multifactorial, caused by synergism with other drugs such as opiates, diffusion hypoxia, and ventilation-perfusion mismatch. In all these other cases, a strong AHVR is very important to mitigate against hypoxaemia. Furthermore, it is now appreciated that relevant concentrations of volatile agents remain in the body for many hours after surgery. Exposure to ~1-2% enflurane or isoflurane for just an hour results in the brain concentrations of these agents remaining >0.1 MAC for several hours [109]. This is concentration which can halve AHVR and at a time when patients are back on the general surgical ward, relatively unsupervised. Part of the hypoxic response also consists of arousal, which itself is a defence mechanism against hypoxia, including that caused by mechanical airway obstruction. In summary, it is for those patients likely at greatest risk of postoperative hypoxemia, such as those with chronic cardiac or lung disease, who we need to adapt techniques to increase safety. It would seem sensible to employ drugs with weakly depressive actions on AHVR (e.g., sevoflurane), rather than those with more strongly depressive actions (e.g., halothane or enflurane). This observation is relevant in diverse situations: isoflurane and sevoflurane have both been proposed as additions to nitrous oxide to enhance analgesia in labour [110, 111]. Pregnant women are clearly at particular risk of hypoxia, so optimal drug combinations with respect to AHVR become very relevant.

Nocturnal hypoxemia can be episodic and associated with tachycardia, myocardial ischaemia, and postoperative myocardial infarction [112]. It is an important research question to establish which anaesthetic combinations worsen or help relieve these episodes [113]. Furthermore, there is a possible interaction of anaesthetics, hypoxia, and thrombosis, with subsequent risks of postoperative deep vein thrombosis or pulmonary embolism [114].

Hypoxia is routinely monitored using pulse oximetry but in fact a pulse oximeter cannot measure minute ventilation. Therefore, Dahan and Teppema proposed that monitoring of PCO_2_ (e.g., using portable, easily applied transcutaneous devices) should become standard practice in recovery rooms and general wards [115].

There is considerable scope for clinical work in the areas indicated, especially to assess whether changes in anaesthetic drugs used or techniques can minimise side effects or indeed postoperative mortality. There remain, however, important gaps in the scientific literature on this subject. This review has, intentionally, focussed upon the role of* volatile *anaesthetics and their effect on the* acute *hypoxic ventilatory response. Perhaps of equal importance is the role of episodic or intermittent hypoxia,* sustained *hypoxia. It is possible that* intravenous *anaesthetics have a very different effect on AHVR [116]. Because they were commonly used only for induction, with anaesthesia subsequently maintained using volatile agents, the practical consequences of intravenous agent effects on postoperative respiratory control were somewhat limited. However, now total intravenous anaesthesia (usually using propofol) is now very common [117].

## 8. Directions for Future Research

The interaction of subject arousal, hypoxia, and anaesthetic agents can be investigated by brain imaging techniques (e.g., functional magnetic resonance imaging, fMRI) which could reveal the sites of action for anaesthetics, and where they might interact with the hypoxic chemoreflex and/or the arousal pathways.

The basic science research focussing on isolated carotid body function can be extended to molecular investigations of how the carotid body transduces the hypoxic signal and how anaesthetics interact with this process.

These researches into actions of anesthetics on cellular/molecular components of the carotid body response might reveal insights that could be exploited for harnessed to therapeutic benefit. For example, if a particular protein channel is discovered responsible for conferring susceptibility to depressive effects of anaesthetic, then identifying it (or its associated gene) might also reveal those patients at greater risk of the depressive response to anaesthetic. Or, this knowledge might enable drug development such as specific anaesthetics that avoid the risk of respiratory depression.

## Figures and Tables

**Table 1:** Summary of studies examining effect of volatile anaesthetics on carotid body responses.

Study	Agent	Number of animals	Dose (%)	Dose (MAC)	NMB	Method	Animal	Anaesthetic effect for hypoxic response	Anaesthetic effect for hypercapnic response
Biscoe and Millar 1968 [56]	Halothane	1	1.00	1.250	Y	IA-CSN	Cat	1.04	—
Biscoe and Millar 1968 [56]	Halothane	1	2.00	2.500	Y	IA-CSN	Cat	0.69	0.50^¶^
Davies et al. 1982 [57]	Halothane	6	0.50	0.625	Y	IA-CSN	Cat	0.58	0.49
Berkenbosch et al. 1982 [63]	Halothane	2	0.80*	1.000	N	ABP	Cat	—	0.58
Berkenbosch et al. 1982 [63]	Halothane	3	1.00*	1.250	N	ABP	Cat	—	0.50
van Dissel et al. 1985 [62]	Halothane	6	1.00*	1.250	N	ABP	Cat	0.54	0.68
Morray et al. 1996 [60]	Halothane	6	0.50	0.625	Y	IA-CSN	Cat^†^	1.84	—
Morray et al. 1996 [60]	Halothane	6	1.00	1.250	Y	IA-CSN	Cat^†^	0.20	—
Ide et al. 1999 [58]	Halothane	10	0.10	0.125	Y	CBP-PN	Cat	0.94	—
Ide et al. 1999 [58]	Halothane	10	0.80	1.000	Y	CBP-PN	Cat	0.11	—
Ponte and Sadler 1989 [61]	Halothane	5	0.75*	0.938	Y	IA-CSN	Rabbit	1.00	1.17
Ponte and Sadler 1989 [61]	Halothane	5	0.75*	0.938	Y	IA-CSN	Cat	0.92	1.08
Ponte and Sadler 1989 [61]	Enflurane	4	0.80*	0.500	Y	IA-CSN	Rabbit	0.67	0.96
Ponte and Sadler 1989 [61]	Enflurane	7	0.80*	0.500	Y	IA-CSN	Cat	0.96	1.00
Ponte and Sadler 1989 [61]	Isoflurane	4	0.75*	0.682	Y	IA-CSN	Rabbit	0.89	0.88
Ponte and Sadler 1989 [61]	Isoflurane	8	0.75*	0.682	Y	IA-CSN	Cat	0.67	0.96
Joensen et al. 2000 [59]	Isoflurane	3	0.10	0.091	N	IA-CSN	Rabbit	0.69	0.75
Joensen et al. 2000 [59]	Isoflurane	6	1.00	0.909	N	IA-CSN	Rabbit	0.92	1.75

The order of studies in the table is chronological for each agent studied. The columns show the agent used, the number of animals studies, the dose in % and then in MAC, whether (Y) or not (N) neuromuscular blockade was used, the experimental preparation, the animal species, the standardised anaesthetic effect for hypoxic response, and the standardised anaesthetic effect for hypercapnic response. The experimental preparations were (column 7) intact animal, recording from carotid sinus nerve (IA-CSN), artificial brain perfusion which separates the central from peripheral respiratory structures (ABP), and separate carotid body perfusion with recording from phrenic nerve (CBP-PN). *Only a dose range was specified, and in these cases we have used the middle of the reported range for our calculations (see text). We took the MAC for halothane to be 0.8%, for enflurane to be 1.6%, and for isoflurane to be 1.1%. ^†^Kittens were used. ^¶^The dose of halothane for the hypercapnic study was 2.5% (3.125 MAC).

**Table 2:** Ranking of studies by quality (the Berkenbosch study was not ranked as it examines hypercapnia).

Study	Rank quality of study
Biscoe and Millar 1968 [56]	7
Biscoe and Millar 1968 [56]	7
Davies et al. 1982 [57]	2
Berkenbosch et al. 1982 [63]	—
Berkenbosch et al. 1982 [63]	—
van Dissel et al. 1985 [62]	3
Morray et al. 1996 [60]	5
Morray et al. 1996 [60]	5
Ide et al. 1999 [58]	1
Ide et al. 1999 [58]	1
Ponte and Sadler 1989 [61]	4
Ponte and Sadler 1989 [61]	4
Ponte and Sadler 1989 [61]	4
Ponte and Sadler 1989 [61]	4
Ponte and Sadler 1989 [61]	4
Ponte and Sadler 1989 [61]	4
Joensen et al. 2000 [59]	6
Joensen et al. 2000 [59]	6
